# Loneliness in Older Indian Dyads

**DOI:** 10.3390/ijerph19095302

**Published:** 2022-04-27

**Authors:** Aparna Shankar, Tara Kidd

**Affiliations:** 1 Department of Psychological Sciences, FLAME University, 401 Phoenix Complex, Bund Garden Rd., Opp. Residency Club, Pune 411001, India; 2 Department of Psychology, Liverpool John Moores University, Liverpool L3 3AF, UK; t.m.kidd@ljmu.ac.uk

**Keywords:** loneliness, physical functioning, cognitive functioning, ageing

## Abstract

Background: Loneliness has been recognised as a major public health concern in older adults in developed nations, with little focus on low- and middle-income countries such as India. While the protective nature of social relationships on loneliness has been explored in the context of marriage, typically these benefits are examined in individual spouses rather than within the marital dyad. Methods: A sample of 398 opposite-sex married Indian couples (mean age 54.8 years) was obtained from the pilot wave of the Longitudinal Aging Study in India (LASI) conducted in 2010. These cross-sectional data were analysed using the Actor-Partner Interdependence Model, with one’s own and one’s partner’s cognitive function, functional limitations, depressive symptoms, employment status and contact with friends included as predictors of loneliness. Results: There were no gender differences in the pattern of associations. Depression was positively associated with loneliness with actor and partner effects being significant. One’s partner being employed was associated with less loneliness. Conclusions: The sample showed low levels of depression, loneliness, and reduced functionality; however, depression still predicted one’s own and one’s partner’s loneliness. Future work using longitudinal data could examine the role of employment in loneliness, particularly within the context of gender roles.

## 1. Introduction

Loneliness or ‘perceived social isolation’ is an unpleasant emotional state caused by dissatisfaction with one’s social relationships [1]. Loneliness is a subjective experience and can be distinguished from more objective measures of social relationships that include social isolation, social network size, frequency of contact with family or friends and participation in social activities [2]. It has been recognised as a major public health concern, given its deleterious effects on physical and mental health [3]. While loneliness affects individuals of all ages, a substantial body of research has focused on loneliness in later life [4,5]. Studies find that loneliness is common as people age [4] and that it has a significant impact on health and wellbeing among older adults [6,7].

The majority of research on loneliness has focused on ageing populations in developed nations with relatively limited data available on loneliness in low- and middle-income countries [4]. This may, to some extent, reflect different demographic patterns as the USA, many parts of Europe, and Japan have a significantly larger ageing population when compared with countries such as India. As of December 2020, under 7% of Indians were aged 65 years and over. However, sustained declines in birth rates combined with increasing life expectancy mean that India has seen a steady increase in the number of older adults. Current projections suggest that adults aged 60 years and over will constitute over 13% of the population in India by 2031 [8], and by 2050, India will have over 500 million adults aged 60 and over [9]. Hence, understanding the lives of older adults in India, particularly in the context of changes to the traditional family structure, is extremely important. Moreover, significant changes to behaviours and health begin to take place well ahead of retirement. For instance, chronic health conditions develop earlier in Indians when compared to Western populations, and as a result, ageing studies in India begin at an earlier stage than many US or European studies [10,11]. Given the strong links between these variables and loneliness [12], it is important that individuals are examined in advance of retirement to enable better understanding of future predictors of loneliness.

Recent work using the first wave (2017–2018) of the Longitudinal Ageing Study in India (LASI) found that 20.5% of adults aged 45 years and over in India reported moderate loneliness, while 13.3% reported severe loneliness [13]. Data from the 10/66 Dementia Research Group on adults aged over 65 years from one rural and one urban location in India (*n* = 1001 at baseline) found that 18.3% of participants reported feeling lonely [14]. Thus, loneliness does appear to be a significant issue among older Indians. Further research is essential in understanding loneliness in countries such as India where there is a predominantly young population at present but where the ageing population is expected to grow rapidly.

There is overwhelming evidence that across the life course, close supportive relationships enhance health and well-being [15,16,17]. A key predictor of loneliness in older adults is relationship status; being single, widowed or divorced is a strong predictor of loneliness [14,18]. Loneliness is, however, also prevalent among individuals who are partnered [19]. Shrinking social networks in later life may mean that the relationship with one’s spouse becomes more central [20]. Correspondingly, poor marital quality and dissatisfaction are associated with greater loneliness [19,21,22], whereas high levels of satisfaction may protect against loneliness in older adults [23].

Loneliness has been identified as a determinant of cognitive decline and dementia [24], as well as of poor physical functioning and disability [25,26] among older adults. Interestingly, studies indicate that these associations may be bidirectional, with poor cognition and physical function leading to increases in loneliness over time [27,28,29]. Similarly, loneliness is associated with an increased risk of developing depression [30], with evidence suggesting that this association may also be bidirectional [31]. Indeed, a review by Cohen-Mansfield et al. identifies poor health, poor functional status, worse mental health and cognitive problems as important determinants of loneliness among older adults [12].

Surprisingly, scant research has been conducted on loneliness and its correlates in married older dyads [32]. The majority of research cited above uses the individual as the unit of analysis. This fails to take into account the interdependence of the marital relationship, that partners’ emotions and behaviours influence each other [33], and such effects may be even more marked in stable, older couples. While one’s own health and functioning may impact loneliness, one’s partner’s health may also play a significant role in one’s own well-being. Wong and Hsieh, in their dyadic analysis of older American couples, found that wives reported more strain and less support in their relationships with their husbands when the husbands had any functional limitations. Husbands did not, however, show this pattern when their wives had functional limitations. Husbands reported less strain in other relationships when their wives had limitations, but greater strain when they themselves had limitations [34]. The same study also found no effect of one’s own or one’s partner’s cognitive limitations on one’s own or partner’s reports of marital quality; however, women whose partners reported cognitive problems reported greater support from family and friends [34]. The authors interpret these findings with respect to support received by individuals whose partners may have physical or cognitive limitations. They also highlight the gender differences in caring responsibilities, which leads to women experiencing more marital strain when husbands are unwell. Other work examining couples has found increases in loneliness associated with development of cognitive impairment or dementia in one’s partner [35]. Findings from a Dutch study of older adults shows that spousal disability was associated with increased emotional loneliness among both men and women [36]. Other aspects of health, including one’s own and one’s partner’s mental health may also affect individual loneliness. A considerable body of research has shown concordance in couples’ mental health [37,38,39].

The wider social network and social connections are also an important determinant of loneliness. While contact with children, grandchildren and other relatives might be similar for both members of the dyad, frequency of contact with friends may differ. Ermer and colleagues found increased frequency of contact with friends to be protective against loneliness for wives in older American couples [40,41]; no such association was found for husbands. Supportive friendships may also attenuate the influence of negative marital interactions on loneliness [42], although the evidence is mixed [32]. Workplace colleagues are often an important part of one’s social network. Changes to the global patterns of work mean that fewer people are exiting the labour market prior to retirement; indeed, there is an increase in the proportion of those working past retirement age [43,44]. Being employed is associated with greater financial security, with financial insecurity associated with higher levels of loneliness in older adults, including those who are retired [45,46].

The present study aims to examine predictors of loneliness among older Indian adults. In particular, the study uses the Actor-Partner Interdependence Model [47] to assess how an individual’s own physical health, cognitive function, mental health, contact with friends, and employment status affects their own levels of loneliness as well as that of their partner. The analyses will also examine if these associations differ based on gender. Based on previous research, we hypothesise that husbands’ physical and cognitive function will be associated with their own loneliness as well as their wives’ level of loneliness, while wives’ physical and cognitive function will be associated with their own loneliness but not that of their husbands. Both employment and contact with friends are expected to be associated with one’s own level of loneliness with stronger effects of contact with friends seen for wives. Depression is expected to affect one’s own as well as one’s partner’s level of loneliness.

## 2. Methods

### 2.1. Participants

A cross-sectional design was used with data obtained from the pilot wave of the Longitudinal Aging Study in India (LASI) conducted in 2010 across four Indian states (Kerala, Karnataka, Punjab and Rajasthan). The sample included 1683 adults aged 45 years and over. Data were also collected on spouses (including those who were under the age of 45 years) who resided at the same address and who consented to participate. More information on the sample, method and measures for this wave is available elsewhere [48]. The LASI study was designed to be comparable with other ageing studies around the world such as the Health and Retirement Study (HRS) [49] and the English Longitudinal Study of Ageing (ELSA) [50]. The present analysis includes 398 opposite-sex married couples with at least one partner aged 45 years, who completed the survey in person and had complete data on all variables of interest. Participants were excluded if (a) they were married but data for their spouse was unavailable as the spouse either did not live with them or had not consented to participate (*n* = 155), (b) they were separated, divorced, widowed or never married (*n* = 320) or (c) they had missing data on any of the variables included in the analysis (*n* = 412).

### 2.2. Measures

*Loneliness* was assessed using the short form of the revised UCLA Loneliness Scale [51]. The scale consists of 3 items with response options *hardly ever or never, some of the time* and *often.* Responses to the items are summed to obtain the total scale score which ranges from 3 to 9 with higher scores indicating greater levels of loneliness (α = 0.72).

*Physical function* was assessed by asking participants whether or not they had difficulties with carrying out six activities of daily living, namely bathing, dressing, eating, getting in and out of bed, walking across the room and using the toilet. Responses were summed, and the total score was then dichotomised to indicate if the participant had difficulties with any activities of daily living or not.

*Cognitive function* was assessed by using a test of *memory* (recall) and of *executive function* (verbal fluency). Recall was assessed using a measure that forms part of the adapted Telephone Interview for Cognitive Status [52] used in the Health and Retirement Study [53]. Participants were presented with a list of 10 words, following which they were asked to recall as many as they could (immediate recall). Participants were also asked to recall these words after a short interval (delayed recall). The sum of both recall scores was used as a measure of memory. Total scores could range from 0 to 20, with a higher score indicating better recall. Verbal fluency was assessed using an oral animal naming test which forms part of various tests including the Western Aphasia Battery and the Boston Diagnostic Aphasia examination [54]. Participants were asked to name as many animals or birds as they could in a one-minute interval. Similar measures of recall and verbal fluency are used in other ageing studies such as the HRS and ELSA.

*Depressive symptoms* were measured using the 8-item Centre for Epidemiologic Studies—Depression scale [55,56], which asks the participants to report the frequency with which they experience symptoms of depression with response options *rarely or none of the time, some or a little of the time*, *occasionally or a moderate amount* and *most or all of the time.* The total score for the CESD was calculated excluding the item on loneliness, and ranged from 0 to 21, with higher scores indicating greater depressive symptoms. The 8-item CESD has been shown to be a valid and reliable measure for screening for depression among older adults [57,58].

*Contact with friends* was assessed by asking participants if they had any friends. Those who replied in the affirmative were asked how often they meet up with friends, how often they speak on the phone with their friends and how often they write or email their friends. Each item had the following response options: *never*, *less than once a year, once or twice a year*, *every few months, once or twice a month, once or twice a week, three or more times a week* and *daily*, scored from 0 to 7. Responses on the 3 items were summed and participants who reported having no friends were given a score of 0. Total scores ranged from 0 to 21, with higher scores indicating more frequent contact with friends. As over half of the participants had a score of 0 on this variable, it was dichotomised to indicate no contact versus at least yearly contact.

*Employment status* was classified as currently in work or not.

*Covariates* included participants’ age, ever having a diagnosis of any of the following chronic health conditions (high blood pressure, diabetes, cancer, lung disease, heart problems, stroke, arthritis or psychiatric problems) versus not, and quartiles of total household income.

### 2.3. Statistical Analysis

**Figure 1:**
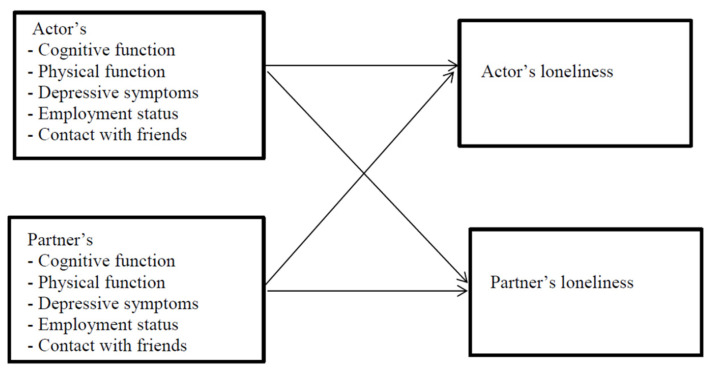
Actor-Partner Interdependence Model.

Descriptive statistics (means and standard deviations for continuous variables, frequency for categorical variables) are reported separately for husbands and wives. Age was mean-centered. Data were analysed using the Actor-Partner Interdependence Model (APIM) [47]. This approach allows us to account for the interdependence in couples’ loneliness [59]. The model includes predictors for the individual as well as their partner, thereby enabling us to estimate the effect of the individual’s own score on the predictor on the outcome and also the impact of the partner’s score on the predictor variable on the outcome. Interactions with gender were added to the model to assess if the associations differed for husbands and wives. Analyses were adjusted for age, presence of any chronic health condition and quartiles of household income. Figure 1 indicates the model that was tested in our analyses. All analyses were carried out using IBM SPSS v.28 (Armonk, NY, USA; IBM Corp.). The APIM was fitted using the MIXED command in SPSS with the dyad as the subject, with unstandardized regression coefficients (B) and corresponding standard errors for the effects reported. We also report R^2^ for the full model [60].

## 3. Results

When compared with participants included in the analyses, those excluded had higher levels of loneliness (mean scores 4.4 vs. 3.8, *p* < 0.001) and depression (mean 3.9 vs. 3.1, *p* < 0.001). They were also, on average, older (mean age 56.3 years vs. 54.8 years, *p* < 0.01), with poorer levels of recall (mean total recall 8.4 vs. 9.3, *p* < 0.001), and a higher proportion had at least one physical limitation (15.4% vs. 10.2%, *p* = 0.002). There were, however, no significant differences between the groups in verbal fluency (mean number of animals named by those excluded was 9.5 vs. 9.9 for those included in the analysis, *p* = 0.202). Similarly, those excluded did not differ from those in the analytic sample on the proportion who had at least one chronic health condition (31.8% vs. 30.5%, *p* = 0.582), at least yearly contact with friends (43.4% vs. 43.1%, *p* = 0.915), proportion of those in work (14.8% vs. 18.3%, *p* = 0.053) or household income (median Rs. 45,000 vs. Rs. 50,880, *p* = 0.082).

Table 1 provides descriptive statistics for the analytical sample. Husbands were, on average, just under 58 years of age while wives were just under 52 years of age. The mean recall score was over 9 (out of 20) for both groups. Around 10% of the participants in both groups reported difficulties with at least one activity of daily living. The mean score on the CESD was just over 3 for both groups. The mean score on the UCLA loneliness scale was also just over 3 for both groups. Over half of husbands and a third of wives reported contact with friends, while over a quarter of men and 10% of women were currently working. Under a third of husbands and wives reported at least one chronic health problem.

Interactions with gender for actor and/or partner effects of the predictor variables were found to be non-significant, suggesting that the pattern of associations was the same for husbands and wives. Table 2 indicates the pattern of associations for the predictor variables with loneliness for the combined model (over men and women). The R^2^ for the full model was 19.3% (χ^2^ (16) = 137.179, *p* < 0.001). Neither actor nor partner effect was significant for the presence of at least one functional limitation or for contact with friends. Both actor and partner effects for recall were similar (B = 0.025 for actor effect and B = 0.021 for partner) and were non-significant. One’s own and one’s partner’s verbal fluency was also not significantly associated with loneliness. Depression was positively associated with loneliness, with actor and partner effects being significant. An increase in one’s own depressive symptoms was associated with greater loneliness (B = 0.122, *p* < 0.001). The effect of greater depressive symptoms in one’s partner, although not as strong, was also associated with more loneliness (B = 0.071, *p* < 0.001).

Contact with friends was not associated with levels of loneliness with both actor and partner effects being non-significant. The actor effect of being in work was non-significant. One’s partner being employed was, however, associated with less loneliness (B = −0.111, *p* < 0.05).

## 4. Discussion

The present study used the APIM to examine predictors of loneliness among older Indian dyads. In line with previous work on ageing dyads based in the US and Europe with older samples, mean levels of loneliness were low among husbands and wives and there were no significant gender differences in predictors of loneliness across the dyad [23,61]. Depression strongly predicted one’s own and one’s partner’s loneliness, while having a partner who was currently employed was associated with lower levels of one’s own loneliness. Neither measures of physical and cognitive function nor contact with friends was associated with either one’s own or one’s partner’s loneliness.

Consistent with the literature, depression was a significant determinant of one’s own and one’s partner’s level of loneliness [62]. Increased feelings of loneliness are often a feature of depression [31]. Other aspects of depression, including increased social withdrawal, loss of interest, and irritability, are likely to affect the interactions between partners, leading to greater strain in the marriage [63,64] and thereby increased feelings of loneliness. Although levels of depression were low in this cohort, efforts to treat depression that consider the dyad are likely to be beneficial in improving mental health and reducing loneliness in later life [38].

Unemployment has been associated with greater loneliness, particularly among younger age groups [65]. Our study showed that one’s partner’s being employed was associated with less loneliness. In addition to the financial costs of unemployment which may lead to greater loneliness, there may be significant social costs. Previous research using data from the Korean Labour and Income Panel Survey found that wives whose husbands were unemployed reported lower levels of well-being, which was in part explained by factors such as dissatisfaction with personal and social relationships [66]. The impact of one’s partner’s unemployment on one’s own wellbeing has been noted in a number of studies, with effects typically being more marked for women [67,68,69]. In our sample, one’s partner’s employment may account for greater financial and social security, leading to lower levels of loneliness. We believe this finding has significant implications for loneliness in later life, particularly given changing patterns of employment across the world. Recent work suggests that a high proportion of adults aged 60 years and over (i.e., post-retirement age) are working both in India and globally [43,44]. Financial constraints and/or inadequate pension provision often contribute to an inability to exit the workforce in this group [70,71]. Studies also project increases in employment rates in older Indians, particularly those who live in urban areas [72]. Contrary to previous research, we did not find an effect for one’s own unemployment on levels of loneliness. Data from Germany show that unemployment allows individuals to spend more time on leisure activities, which can be considered a positive aspect of unemployment [68] and may to some extent offset feelings of loneliness.

Neither measure of cognitive function was associated with either one’s own or one’s partner’s level of loneliness. In addition, neither one’s own nor one’s partner’s functional limitations were associated with levels of loneliness. Previous work has shown both cognitive function, particularly recall, and functional limitations to be a determinant of future loneliness in individual models [73,74]. Given the increased employment rates in older and post-retirement adults, future research/cohort comparisons may wish to consider the potential protective effects of employment on cognitive function [75], and their joint impact on loneliness. The level of physical impairment was low in this sample, with a very small proportion reporting difficulties with multiple activities of daily living which may not be associated with a significant caregiving burden. Thus, the impact on one’s own and spousal levels of loneliness may be limited. Furthermore, while just under a third of participants in this sample reported a chronic condition, in the majority of cases this was a diagnosis of hypertension (~18% of participants), which is less likely to be associated with significant impairment or limitations.

Loneliness was also not related to either one’s own or one’s partner’s contact with friends. A very high proportion of the analytical sample, particularly women, reported having no friends or no contact with their friends. It has been posited that friendship may be particularly beneficial for those experiencing marital strain in ameliorating loneliness [42]. Marital strain was not accounted for in the analysis and so friendship effects may not have been revealed. However, it must be noted that some studies have also reported that the effects of marital dissatisfaction on loneliness were not attenuated by the presence of other supportive relationships [32]. An alternate explanation is that older Indian adults may spend more time with family members or their spouse [76]. Consequently, frequency of contact with and quality of these familial ties may be more important in determining older adults’ feelings of loneliness than external friendships. Previous research from Europe also suggests that lack of family contact, rather than friends, may be a stronger determinant of loneliness in collectivistic societies [77].

### Strengths and Limitations

It has been noted in the literature that more cross-cultural and longitudinal analyses of predictors of loneliness in older marital dyads are urgently needed [78]. We were not able to fully address these recommendations, as this study reported on cross-sectional data, limiting the future applications of these findings for this relatively “young” older population. The study was able to examine the role of cognitive function, physical function, mental health and social contact on loneliness in older Indian dyads. Although the data used in these analyses are from 2010, and we might expect changes in several of the variables examined here over time, it is expected that the pattern of associations, such as lower depression and loneliness in marital dyads, will remain the same.

While the focus of these analyses was primarily on health and social contact variables, future analysis could focus on the role of marital quality and closeness. Indeed, the cumulative effect of partnership quality on loneliness may become more apparent over time in established dyads, with the increased likelihood of one of more members experiencing financial, physical, or mental health challenges [79]. Additional measures that should also be considered are household composition, particularly given that a large number of Indians may live with children or other family members in later life [76]. The role of labour division could also be explored further in light of our findings regarding employment. This is because unemployment may be associated with more leisure time among men, but for their partners this may mean significant increases in household and care responsibilities, particularly if the husband is unemployed due to poor health or if unemployment leads to significant changes in lifestyle due to financial insecurity. Previous work with a German cohort indicated decreases in satisfaction with housework, leisure and health among women whose partner was unemployed, but the converse was not true [68]. Our study also did not include a measure of social network such as the Lubben Social Network Scale [80], which would enable us to examine the contact and quality of relationships with family and other relatives. This may be particularly important in collectivistic societies such as India.

The current study did exclude participants with missing data on any of the variables used in the analysis and dyads were recruited from four states only, so the resulting sample is unlikely to be representative of the married older adult population across India. Indeed, our sample reported significantly better mental and physical health when compared with participants who were excluded from the analyses. Our sample is also younger than samples from other ageing studies. However, it must be noted that the LASI study starts at age 45 in line with other Asian studies of ageing, and retirement age in India is currently lower than in many Western countries. Furthermore, onset of many chronic conditions begins at an earlier age in the Indian population [11]. Other considerations related to generalisability that have been identified by authors such as Surkalim and colleagues may be the cross-cultural adaptation of standardised loneliness tools. On the one hand, it can be considered a strength of the study that comparative (global) measures have been incorporated to enable direct comparisons between India and other countries, something that is severely lacking in the research literature, while on the other hand, it could be argued that culturally appropriate measures of loneliness are needed and that we must step away from the belief that measures developed in individualistic countries accurately reflect those of collectivist countries. Identifying cultural differences in perceptions of loneliness could be operationalised using co-creation methodologies to understand what loneliness means, and how it is conceptualised within social networks in different communities [4]. This would enable us to understand both risk and protective factors associated with loneliness from a global perspective.

## 5. Conclusions

This study furthers our understanding of the prevalence and correlates of loneliness in older Indian dyads, a population that has received relatively little attention in the literature. In line with the literature reported from developed nations in both older and younger cohorts, depression predicted loneliness. However, in contrast to work with older dyads in the US, measures of physical and cognitive function were not associated with one’s own or one’s partner’s loneliness. The dyads in our sample showed low levels of depression, loneliness and functional limitations. It is our hope that the results from this study will generate important discussions around the differences in the experience and predictors of loneliness, as well as the similarities, between countries. Future research could also consider measures of the composition and quality of social connections on predicting future loneliness. Only when we can fully embrace both similarities and differences between countries in aging research can we truly further our understanding of the global impact of loneliness.

## Figures and Tables

**Table 1:** Descriptive statistics by participant gender (*n* = 398 dyads).

	Husbands	Wives
Age in years–Mean (SD)	57.9 (10.6)	51.7 (10.2)
Any chronic condition–*n* (%)	126 (31.7)	117 (29.4)
Total recall score–Mean (SD)	9.4 (3.3)	9.2 (3.4)
Verbal fluency–Mean (SD)	10.4 (5.7)	9.5 (4.8)
Having at least one functional limitation–*n* (%)	39 (9.8%)	42 (10.6%)
Depressive symptoms–Mean (SD)	3.0 (2.8)	3.1 (2.9)
Loneliness–Mean (SD)	3.8 (1.2)	3.8 (1.2)
Contact with friends–*n* (%)	214 (53.8%)	129 (32.4%)
Currently in work–*n* (%)	105 (26.4%)	41 (10.3%)

**Table 2:** Dyadic analysis of predictors of loneliness in older adults (*n* = 398 dyads).

	Actor’s Loneliness	Partner’s Loneliness
Total recall score	0.025 (0.013)	0.021 (0.013)
Verbal fluency	−0.007 (0.009)	0.008 (0.009)
Having at least one functional limitation	0.111 (0.068)	0.003 (0.067)
Depressive symptoms	0.122 (0.014) **	0.071 (0.014) **
Contact with friends	−0.029 (0.041)	−0.043 (0.041)
Currently in work	−0.057 (0.052)	−0.111 (0.050) *

** *p* < 0.001, * *p* < 0.05.

## Data Availability

Data for the LASI pilot wave are available to download from the Gateway to Aging website (Available online: www.g2aging.com (accessed on 21 January 2021)).
